# FREQUENCY AND MODALITY OF EXERCISE ON PAIN AND INDEPENDENCE IN ELDERLY INDIVIDUALS WITH OSTEOARTHRITIS: A CROSS-SECTIONAL STUDY

**DOI:** 10.1590/1413-785220253301e280703

**Published:** 2025-02-03

**Authors:** Felipe Marrese Bersotti, Reniery Pereira Da Silva, Angelica Castilho Alonso, Guilherme Carlos Brech, Paula Regina Mendes da Silva Serrão, Ulysses Fernandes Ervilha

**Affiliations:** 1 Universidade de São Paulo, School of Arts, Sciences and Humanities, São Paulo, SP, Brazil; 2 Universidade de Sao Paulo, Faculdade de Medicina, Hospital das Clinicas HC-FMUSP, Laboratório de Estudos do Movimento, Sao Paulo, SP, Brazil; 3 Universidade São Judas Tadeu, Graduate Program in Aging Sciences, São Paulo, SP, Brazil; 4 Universidade Federal de São Carlos, Physical Therapy Department, São Carlos, São Paulo, Brazil

**Keywords:** Pain, Exercise, Aged, Osteoarthritis, Knee, Dor, Exercício Físico, Idoso, Osteoartrite do Joelho

## Abstract

**Background:**

Regular physical exercise promotes pain relief, reducing the central facilitation of pain mechanisms.

**Objective:**

Evaluate the effect of different frequencies of physical exercise (once, twice, or three times a week) on different modalities (aerobic training, stretching training, and strength training), on the pain in the knee joint, and on the level of independence on people with knee osteoarthritis.

**Methods:**

Is cross-sectional and used the STROBE-Checklist: cross-sectional studies. A total of 193 elderly people were evaluated, pain and functional independence were analyzed.

**Results:**

For the pain variable, there was a statistical difference in favor of the intervention in the comparisons control versus strength 1 and 2 times a week and stretching 3 times a week already in the Lawton variable, only the comparison control versus aerobic 1 time a week did not prove to be statistically dignified.

**Conclusion:**

The exercise modality and the weekly frequency seem to affect the perception of pain, stretching exercises performed three times a week, as well as muscle strengthening exercises, regardless of weekly frequency are efficient in joint pain analgesia. Practicing muscle strength exercises, regardless of weekly frequency and aerobic and stretching exercises at least twice a week, increases and/or maintains IADL. ***Level of Evidence II; Cross-sectional Study.***

## Resumo

**Introdução:**

O exercício físico regular promove o alívio da dor, reduzindo a facilitação central dos mecanismos álgicos.

**Objetivo:**

Avaliar o efeito de diferentes frequências de exercício físico (uma, duas ou três vezes por semana) em diferentes modalidades (treinamento aeróbico, treinamento de alongamento e treinamento de força), na dor na articulação do joelho e no nível de independência em pessoas com osteoartrite de joelho.

**Métodos:**

Este artigo é um estudo transversal e utilizou o STROBE-Checklist: estudos transversais. Foram avaliados 193 idosos. Foram analisadas dor e independência funcional.

**Resultados:**

Para a variável dor, houve diferença estatística a favor da intervenção nas comparações controle versus força 1 e 2 vezes por semana e alongamento 3 vezes por semana, já na variável Lawton, apenas a comparação controle versus aeróbico 1 vez por semana não se mostrou estatisticamente diferente.

**Conclusão:**

A modalidade de exercício e a frequência semanal parecem afetar a percepção da dor, exercícios de alongamento realizados três vezes por semana, bem como exercícios de fortalecimento muscular, independente da frequência semanal são eficientes na analgesia da dor articular. A prática de exercícios de força muscular, independente da frequência semanal e exercícios aeróbicos e de alongamento pelo menos duas vezes por semana, aumenta e/ou mantém as AIVD. **Nível de Evidência II; Estudos Transversais.**

## INTRODUCTION

Knee osteoarthritis, a disease characterized by wear and inflammation of the articular cartilage,^1^ is one of the main causes of functional disability in elderly people.^2^ Pain is a frequent symptom in osteoarthritis and strongly impacts daily living tasks.^3^ Inactivity is a known risk factor for osteoarthritis development.^4^ Physical exercise is a key intervention proposed by health professionals for joint pain treatment, stiffness attenuation, weight control, and reducing sedentary behavior in this population.^4^ While literature defines how much weekly physical activity is needed to be considered active and applies these guidelines to knee osteoarthritis,^5^ no study has determined how many weekly sessions of physical activity are necessary to reduce knee osteoarthritis pain. This is important as individuals with osteoarthritis-related pain need a clear starting point to begin regular physical activity, whether once, twice, or more per week.

A useful tool for evaluating autonomy in elderly functional activities is the Lawton scale.^6^ Dependence is a critical health condition in elderly people, implying self-care reliance on others, communities, or institutions. The World Health Organization defines dependence as a state where decreased functional capacity prevents performing basic daily tasks independently.^7^

Evidence suggests physical activity reduces pain perception.^8^ Studies show regular physical exercise alleviates pain by reducing central pain facilitation, increasing serotonin and opioid levels in central inhibitory pathways, and utilizing endogenous inhibitory systems.^8^ These physiological effects highlight the need to determine the optimal exercise dose/response to mitigate pain perception. We hypothesize that higher exercise frequency, regardless of modality, reduces pain and improves independence in individuals with knee osteoarthritis.

This study aimed to evaluate the effect of different exercise frequencies (once, twice, or three times weekly) across modalities (aerobic, stretching, and strength training) on knee pain and independence in people with knee osteoarthritis.

## MATERIALS AND METHODS

This is an observational cross-sectional study. This article used the Strengthening the Reporting of Observational Studies in Epidemiology Checklist: cross-sectional studies. Approved by the research ethics committee of Universidade de São Paulo (CAAE nº 04867418.6.0000.5390). All elders signed the Free and Informed Consent Form.

### Participants

A total of 193 elderly individuals who had engaged in physical activity in a nursing home were selected, along with a group of 25 participants who had not. Participants were randomly assigned using sealed envelopes before exercise sessions by a person external to the study.

The physical activity groups were distributed as follows: (1) Aerobic Training; (2) Stretching Training; (3) Resistance Training. Each modality had a frequency of (a) once a week, (b) twice a week, or (c) three times a week. The control group performed no training.

### Inclusion criteria

For all groups, inclusion required a medical report and radiographic evidence of osteoarthritis (OA) according to the Kellgren and Lawrence scale.^9^ Specifically, the exercise group had practiced physical activity regularly for over a year. The control group had not engaged in physical activity or rehabilitation in the past 12 months. Exclusion criteria included: (I) previous lower limb surgery, (II) fibromyalgia diagnosis, (III) corticosteroid or intra-articular hyaluronic acid use in the past 12 months, (IV) oral anti-inflammatory use in the past 2 months, (V) physiotherapy treatment for spine, hip, or lower limbs in the past six months, (VI) regular walking for 30 minutes or more daily, (VII) heart failure, (VIII) physical dependence.

### Physical activity description

Aerobic training lasted 50 minutes, with a 5-minute warm-up walk. The protocol included: (I) 30-second brisk walks followed by 30 seconds of rest, repeated 3 times; (II) 30-second directional changes followed by 30 seconds of rest, repeated 3 times; (III) 30 seconds of jumping jacks, 3 sets of 8 repetitions, with 30 seconds of rest. Stretching training also lasted 50 minutes, consisting of static lower limb stretches in a seated position. Each muscle group was stretched for 30 seconds with a 30-second rest (knee flexors and extensors, hip adductors, flexors, and extensors).

Resistance training was performed for 50 minutes at 50% of the 1 maximum repetition (MR). A 5-minute warm-up walk preceded 8 to 10 exercises with 3 sets of 8 repetitions, resting 1 minute between sets. Exercises included strengthening of knee flexors, extensors, hip flexors, abductors, elbow flexors, shoulder flexors, and abductors, using ankle weights.

### Procedures

Pain assessment: Knee pain was assessed using the numerical rating scale: “On a scale from 0 to 10, where 0 is no pain and 10 is the greatest pain imaginable, what is your knee pain today?”

The capacity to perform instrumental activities of daily living (IADL) was evaluated using the Lawton Scale, which consists of nine tasks such as phone use, shopping, food preparation, housework, transportation, medication preparation, and financial management. Responses were classified as: [1] performed the activity, [2] performed with help, or [3] did not perform the task.

### Statistical Analysis

Data distribution was initially checked using the Shapiro-Wilk test. The Kruskal-Wallis test (P ≤ 0.05) was applied, followed by Dunn’s post hoc test. Statistical software used was Prisma version 5.0. The Hedges g-statistic^10^ of the independent t-test was applied to calculate effect size, considering different sample sizes. Effect sizes were classified as small (0.20 ≤ g < 0.50), medium (0.50 ≤ d < 0.80), or large (d ≥ 0.80). SPSS v.20 was used for statistical analysis.

## RESULTS

Table 1 shows the characteristics of the participants in each of the training subgroups and the control group.

**Table 1:** Characteristics of the participants. Sample means (standard deviation).

	aerobic training			stretching training			resistance training			group control	between groups difference
	1 x/week	2 x/week	3 x/week	1 x/week	2 x/week	3 x/week	1 x/week	2 x/week	3 x/week		
	22	17	17	15	19	22	17	22	17	25	
age, y	71.9 (7.5)	75.4 (8.2)	69.6 (6.5)	72.3 (4.4)	70.7 (5.4)	72.4 (6.1)	71.5 (6.0)	72.7 (6.2)	72.7 (5.9)	80.3* (5.9)	p<0.05*
weight, kg	70.8 (12.8)	68.4 (10.6)	65.6 (12.8)	76.6 (11.5)	68.8 (11.6)	67.5 (9.5)	70.5 (9.9)	66.5 (10.1)	67.5 (13.0)	66.8 (15.0)	p>0.05
height, cm	159.2 (9.3)	155.6 (7.1)	157.7 (5.5)	161.0 (7.0)	159.0 (8.3)	157.0 (8.6)	159.1 (7.7)	156.4 (8.0)	154.3 (8.6)	154.9 (8.4)	p>0.05
BMI	27.8 (4.0)	28.3 (4.5)	26.3 (4.9)	29.6 (5.3)	27.2 (4.2)	27.4 (3.7)	28.1 (5.5)	27.1 (3.3)	28.4 (5.3)	27.9 (6.5)	p>0.05

p<0.05* = proved to be significantly different from the other groups

The control group is different from the other ones.

**Figure 1:**
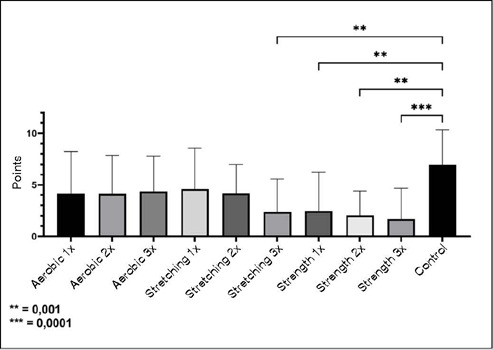
Values of the numeric rating scale acquired from the control group and aerobic training, stretching, and strength groups. 1x, 2x and 3x indicate respectively, one, two, or three training sessions per week.

Figure 1 shows the comparison of pain scale values in the different conditions studied. The group that performed muscle strength exercises once, twice, or three times a week presented lower knee pain compared to the control group (P < 0,001). The group that performed stretching three times a week also reported significantly lower pain scale values when compared to the control group.

Concerning IADL, practicing strength physical exercises at least once a week or stretching or aerobic exercises at least twice a week increases and/or maintains functional independence, when compared to the control group.

Figure 02 - Lawton Scale Variable Comparisons

**Figure 2:**
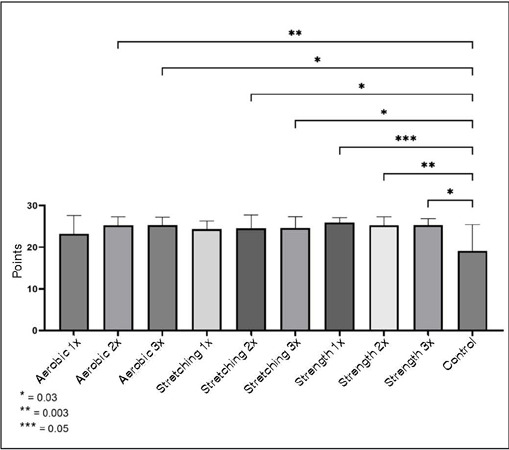
Lawton scale variable comparisons.

Table 2, Table 3 show, respectively, the effect size (size effect) and power effect of the groups when compared to the control group. In contrast the relationship between pain and the Lawton scale.

**Table 2:** Data From Numerical Pain Scale Comparisons.

Comparisons Average (Standard Deviation)			Effect Size (d)	Power Effect
X per week		Control		
**Resistance Training**				
1	2,47 ( 3,76)	6,96 ( 3,36)	1,2733	0,9768
2	2,04 ( 2,35)	6,96 ( 3,36)	1,6738	0,9999
3	1,70 ( 2,97)	6,96 ( 3,36)	1,6361	0,9991
**Stretching Training**				
1	4,60 ( 3,94)	6,96 ( 3,36)	0,6581	0,5018
2	4,15 ( 2,83)	6,96 ( 3,36)	0,8909	0,8158
3	2,36 ( 3,20)	6,96 ( 3,36)	1,3985	0,9967
**Aerobic Training**				
1	4,13 ( 4,09)	6,96 ( 3,36)	0,7585	0,7187
2	4,11 ( 3,73)	6,96 ( 3,36)	0,8082	0,7084
3	4,35 ( 3,44)	6,96 ( 3,36)	0,7680	0,6644

**Table 3:** Data From Lawton Scale Comparisons.

Comparisons Average (Standard Deviation)			Effect Size (d)	Power Effect
X per week		Control		
**Resistance Training**				
1	26,00 ( 1,17)	19,12 ( 6,35)	1,3824	0,9901
2	25,22 ( 2,13)	19,12 ( 6,35)	1,2556	0,9875
3	25,29 ( 1,64)	19,12 ( 6,35)	1,2273	0,9678
**Stretching Training**				
1	24,33 ( 2,05)	19,12 ( 6,35)	1,0023	0,8486
2	24,52 ( 3,25)	19,12 ( 6,35)	1,0289	0,9103
3	24,59 ( 2,78)	19,12 ( 6,35)	1,0908	0,9546
**Aerobic Training**				
1	23,22 ( 4,43)	19,12 ( 6,35)	0,7410	0,6990
2	25,23 ( 2,13)	19,12 ( 6,35)	1,1983	0,9607
3	25,29 ( 1,96)	19,12 ( 6,35)	1,2166	0,9653

## DISCUSSION

The main findings highlight the importance of regular exercise, regardless of type, in managing knee osteoarthritis and improving functional independence. Exercise frequency plays a significant role in its effectiveness. The cross-sectional design limits the ability to establish causation, and sample size should be acknowledged as a limitation. Strength training reduces pain in individuals with knee osteoarthritis through various mechanisms, such as improving muscle strength around the knee, which provides support and stability, reducing stress on the joint. This leads to reduced pain and discomfort.^11^ Additionally, it can improve joint mobility, reduce stiffness, and enhance physical function.^11^,^12^ Strength training also improves bone density, reducing fall and fracture risks.^12^ The frequency of sessions required for analgesic effects remains under study. Our findings align with Jorge et al.^11^, who used a twice-weekly protocol, and Bennell et al.^12^, who recommended three or more sessions a week. However, our study shows that this exercise type promotes analgesia regardless of frequency.

Stretching exercises require at least three weekly sessions for pain relief, as confirmed by Weng et al.^13^, whose eight-week study reduced knee pain in OA patients. Stretching improves joint range of motion and reduces stiffness, contributing to pain relief. The physiological benefits include increased muscle extensibility and reduced muscle stiffness, improving movement and functional synergy. These acute responses are linked to chronic adaptations, such as better joint mobility and flexibility.^13^

Aerobic training, regardless of frequency, did not show significant effects on knee pain compared to the control group. Wallis et al.^14^ also found no positive impact on knee pain, though improvements were observed in cardiovascular health. However, recent studies suggest aerobic exercise can reduce knee pain.^15^ A systematic review by Raposo et al.^16^ showed that aerobic exercise benefits pain reduction. Thus, factors like activity duration may limit the analgesic effects of aerobic training in this study.

While aerobic exercise provides cardiovascular and other health benefits, it may not be as effective in reducing knee pain compared to strength training. Repetitive movements in aerobic activities can stress the knee joint, worsening pain. Aerobic exercises also don’t improve muscle strength and joint stability as effectively as strength training. Some individuals may find aerobic activities too painful, reducing their willingness to participate regularly. While aerobic exercise is beneficial, other exercises like strength training or low-impact activities may be more effective for pain relief.^17^,^18^,^19^,^20^

Although this study offers valuable insights, it only focuses on the role of exercise in pain reduction in knee OA. A more individualized approach, addressing specific patient needs, is required. Interdisciplinary research should explore comprehensive treatment strategies, combining exercise, medication, diet, and lifestyle changes, with potential surgery. Future studies should investigate combined treatment approaches for knee OA.

This study has clinical implications for knee osteoarthritis management, showing that exercise can effectively reduce pain and improve daily function. However, care should be taken when prescribing exercise modalities and frequencies for knee OA patients.

## CONCLUSION

Based on the findings of the study, it can be concluded that resistance training is an effective form of exercise for reducing knee pain and improving functional independence in individuals with knee osteoarthritis. This effect was seen even with a minimal frequency of once a week, although a higher frequency of training (two or three times a week) may have even greater benefits.

Stretching training was found to be effective in reducing knee pain only when performed three times a week, and improved functional independence when done two to three times a week.

Aerobic training did not show significant improvements in pain reduction, but it did have a positive effect on functional independence when performed two to three times a week.
